# Les leishmanioses cutanées à *Leishmania major* et à *Leishmania tropica* au Maroc: aspects épidémio-cliniques comparatifs de 268 cas

**DOI:** 10.11604/pamj.2014.19.160.2613

**Published:** 2014-10-16

**Authors:** Soumia Chiheb, Widad Slaoui, Tarik Mouttaqui, Meriem Riyad, Hakima Benchikhi

**Affiliations:** 1 Service de Dermatologie-Vénéréologie Ibn Rochd de Casablanca, Casablanca, Maroc; 2 Laboratoire de Parasitologie-Mycologie de la Faculté de Médecine de Casablanca, Casablanca, Maroc

**Keywords:** Major, tropica, polymorphisme, Maroc, Major, tropica, polymorphism, Morocco

## Abstract

**Introduction:**

Depuis 1995, le Maroc a connu une réactivation des foyers de leishmanioses cutanées (LC) à *L. major* et une nouvelle répartition géographique des foyers à *L. tropica*. Le but de cette étude est de comparer les aspects épidémio-cliniques associés aux LC potentiellement dûes à *L. major* et à *L. tropica*.

**Méthodes:**

Une étude rétrospective a colligé 268 cas de LC au service de dermatologie du CHU Ibn Rochd de Casablanca entre Janvier 1995 et Septembre 2010. Les données étaient analysées par Epi info version 3.5.1. Le test X2 était appliqué (Différence significative = p< 0,05).

**Résultats:**

Deux cent soixante-huit cas de LC ont été colligés, dont 160 femmes et 108 hommes. Ils ont été répartis en 123 patients originaires des foyers à L.major et 145 patients originaires des foyers à *L. tropica*. L'aspect ulcéronodulaire, ulcérovégétant ou végétant était retrouvé dans 58 cas (47,2%) des cas de LC à *L. major* versus 24 cas (16,7%) dans la L.C à *L. tropica*. L'aspect papulonodulaire était retrouvé dans 84 cas (58%) de LC à *L. tropica* contre 41 cas (33,3%) de LC à *L. major*.

**Conclusion:**

Dans la LC à *L. major*, l'atteinte des membres et les aspects cliniques végétant ou ulcéro-végétant restent toujours prédominants. Dans la L.C à *L. tropica*, l'atteinte papulonodulaire unique du visage reste prédominante mais des formes ulcéronodulaires, végétantes ou ulcérovégétantes existent également dans les foyers récents à *L. tropica*, prêtant à confusion cliniquement avec des LC à *L. major*.

## Introduction

Avant 1995, deux formes nosogéographiques de leishmanioses cutanées (LC) étaient décrites au Maroc. La LC à *L. major* du Sud décrite comme une forme humide, multiple prédominant aux membres inférieurs dans les foyers endémo épidémiques; la LC à *L. tropica* du centre décrite comme de type lupoïde dans les foyers hypo endémique [1]. A partir de 1995, l'apparition de nouveaux foyers épidémiques de LC à *L. tropica* au Nord et au centre sud du pays s'est accompagnée d'un polymorphisme clinique important, prêtant confusion avec une LC à *L. major* [2]. Le but de ce travail est de comparer les aspects épidémio-cliniques des 2 types de leishmaniose à travers l'expérience du service de dermatologie du CHU IBN Rochd de Casablanca.

## Méthodes

L’étude a porté sur 268 cas de LC recrutés entre Janvier 1995 et Septembre 2010. Le diagnostic de LC a été retenu sur la notion d'habitat ou de séjour en zone d'endémie, l'aspect clinique évocateur, la localisation aux zones découvertes, l'absence de guérison des lésions après une antibiothérapie banale, un examen parasitologique direct positif et /ou une histologie évocatrice. Ces dossiers ont fait l'objet d'une analyse épidémioclinique, précisant en particulier l'origine de la maladie et les différents aspects cliniques observés.

La distinction des foyers de LC à *L. major* et à *L. tropica* s'est basée sur la définition de ces foyers à travers les données de la littérature [3] et les données du ministère de la santé publique, elle-même basée sur le biotope, le typage iso enzymatique des parasites ou la biologie moléculaire. Ainsi, nos observations ont été scindées en 2 groupes: Un groupe de LC à *L. major* originaire des foyers du Sud et de l'Est du Maroc; Un groupe de LC à *L. tropica* originaire des foyers du centre, centre sud et Nord.

Pour chaque groupe étaient précisés l’âge, le sexe, la date d'apparition, la durée d’évolution, les aspects cliniques, la localisation des lésions, leur taille, l’évolution après traitement. Les aspects cliniques évocateurs retenus étaient la forme papulonodulaire, végétante, ulcéro-nodulaire ou ulcéro-végétante, et ulcéro-croûteuses. La fréquence de ces aspects cliniques a été comparée dans les foyers supposés à *L. major* et dans les foyers supposés à *L. tropica*. Les données étaient analysées par Epi info version 3.5.1. Le test X2 était appliqué et les différences étaient significatives quand p. était inférieur à 0,05.

## Résultats

Sur une période de 15 ans, 268 cas ont été diagnostiqués. Ils se répartissaient en 123 patients originaires des foyers du Sud et de l'Est du Maroc potentiellement dus à *L. major* et 145 patients des foyers du Nord, Centre et Centre-Sud potentiellement dus à *L. tropica*. La moyenne d’âge des patients issus des foyers de L.C à *L.major* était de 33,5 ± 23,1 ans avec des extrêmes de 1 à 84 ans, et celle des patients issus des foyers *L. tropica* était de 26,6 ±21,65 ans avec des extrêmes de 1 à 72 ans.

Le sexe féminin était prédominant dans les LC à *L. tropica*: 61,4% versus 38,6% avec p < 0,05. La durée d’évolution des lésions cutanées de la LC à *L. major* était de 4,5±7,3 mois tandis que celle de la L.C à *L. tropica* était de 9,1 mois ±10, 3 mois. La localisation anatomique est rapportée dans leTableau 1, on note: L'atteinte des membres est retrouvée dans 72 cas (61,7%) de leishmanioses à*L. major*versus 28 cas (19,3%) dans la LC à *L. tropica* avec une différence significative (P < 0,05); L'atteinte du visage est retrouvée dans 102 cas (70, 3%) de LC à *L. tropica* avec une différence très significative (p < 0,05).

**Tableau 1:** Localisation des lésions de LC à L.major et à L.tropica

		*L.major*	*L.tropica*	*P Value*
Localisation	Visage	29(23,6)	102(70,3)	0,00000000
	Membres	72(61,7)	28(19,3)	0,00000000
	Visage et membres	18(14,6%)	12(8,27%)	0,009
	Tronc	0	3(2,08%)	0,1
	Total	123	145	

Le nombre moyen de lésions dans la LC à *L. major* était de 3,1 ± 2,7, avec un maximum de 14. Les lésions étaient multiples dans 61% des cas. Le nombre moyen de lésions dans la LC à *L. tropica*était de 1,5 ± 1,1, avec un maximum de 8; la lésion était unique dans 74% des cas.

Le diamètre des lésions variait de 1 à 15 cm dans la LC à L.major avec une moyenne de 3,8±2,3 cm. Les lésions variaient de 1 à 10 cm dans la LC à *L. tropica*, avec une moyenne de 2,3 ±1,6 cm.

**Figure 1:**
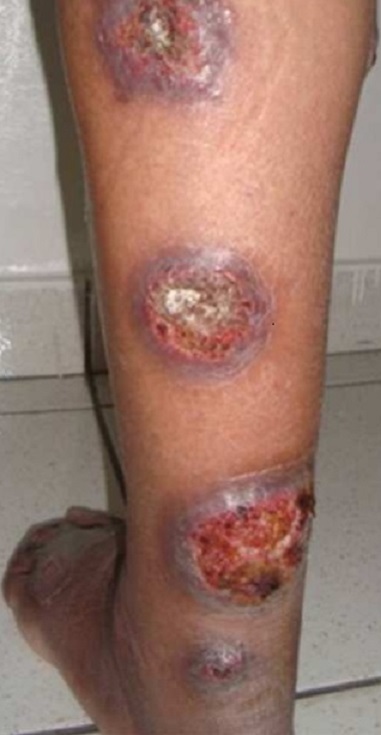
Lésions verruqueuses et ulcérées du membre inférieur (L. major)

**Figure 2:**
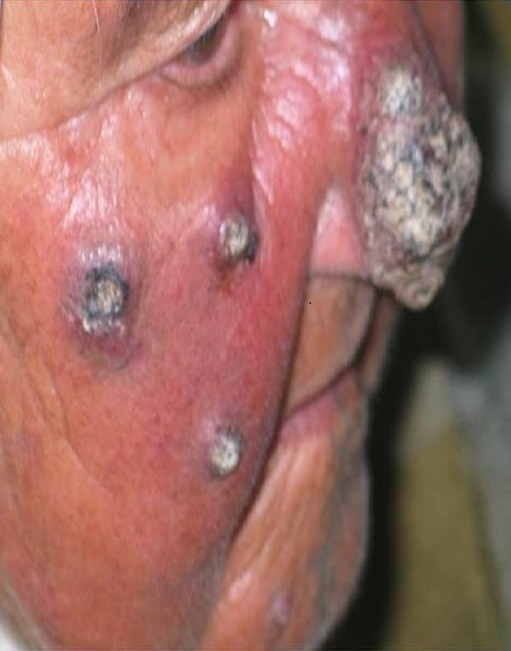
Lésions végétantes multiples atypiques du visage (L. tropica)

L'aspect ulcéronodulaire, ulcérovégétant ou végétant est retrouvé dans 58 cas (47,2%) des de LC à*L. major* (Figure 1) versus 24 cas (16,7%) des L.C à *L.tropica* (Figure 2). L'aspect papulonodulaire était retrouvé dans 84 cas (58%) des LC à *L. tropica* contre 41 cas (33,3%) de LC à*L. major*. (p: 0,000005) (Tableau 2).

**Tableau 2:** Aspects cliniques des LC à *L.major* et à *L.tropica*

		LC à L. major	LC à L. tropica	P
Aspects cliniques	Papulo-nodulaire	41(33,3%)	84(58%)	0,00005
	Végétant ou verruqueux	21(17,1%)	10(7%)	0,009
	Ulcéro-nodulaire ou ulcéro-végétant	37(30,1%)	14(9,7%)	0,0002
	Ulcéro-croûteux	12(9,8%)	26(18%)	0,055
	Cicatriciel	5(4,1%)	4(2,8%)	0,55
	Autres (Erythème pur, sporotrichoide)	7(5,7%)	7(4,8%)	
	Total	123	145	

## Discussion

La LC à *L.major* classiquement décrite comme une forme humide, végétante ou ulcéro-végétante prédominant aux MI est également vérifié dans cette étude [4].

Par contre la L.C à *L. tropica* décrite comme une forme sèche papulonodulaire unique du visage dans les foyers hypo endémiques stables, connait actuellement [5–7] un polymorphisme clinique important dans les foyers épidémiques récents à L.tropica [8, 9]. La forme papulonodulaire du visage reste prédominante: 84 cas (58%) mais d'autres aspects cliniques telle que la forme ulcéro-nodulaire, ulcéro-végétante ou végétante mimant une LC à *L. major*sont actuellement retrouvés dans les LC à *L. tropica* sans différence significative par rapport à *L.major*.

La distinction des formes cliniques à *L. major*ou à *L. tropica* s'est basée, dans notre étude, sur la corrélation entre l'origine géographique des cas et la définition des foyers comme étant à *L. tropica*ou *L. major*par le ministère de la santé publique et les données de la littérature.

L'origine géographique des patients avait montré une diversité dans les foyers potentiellement dus à*L. tropica*, regroupant les provinces du Centre, Centre Sud et Nord du pays, tandis que la LC à *L.major* est restée cantonnée au Sud et à l'Est [3, 10].

L'analyse des résultats montre une prédominance féminine dans les foyers à *L.tropica* liée d'une part au caractère endophile du Phlebotomus sergenti [11] (les femmes passant la nuit à l'intérieur des maisons par rapport aux hommes, et d'autre part l'atteinte prédominante du visage engageant le pronostic esthétique justifie une consultation fréquente des femmes.

Dans les foyers de LC à *L. major*, l'atteinte des 2 sexes ne montrent pas de différence significative, par contre l'atteinte des membres est prédominante par rapport aux foyers à *L. tropica* (P < 0.05).

Les formes, du sud et de l'est du Maroc à *L. major*, sont caractérisées par un mode d’évolution aigu avec une durée moyenne d’évolution des lésions de 4,5 mois dans notre étude [12].

Tandis que la LC à *L. tropica* évolue sur un mode chronique avec une durée d’évolution plus longue, de 9,1mois en moyenne; cette durée d’évolution est classiquement supérieure à 2 ans dans les foyers d'endémicité stable [13].

Les lésions sont, le plus souvent, multiples dans la LC à *L. major* atteignant un nombre moyen de 3,1 pour chaque patient dans notre travail; versus 1,5 pour la L.C à *L.tropica* où 74% des patients ne présentaient qu'une seule lésion. L'analyse de contenus stomacaux des phlébotomes montre la présence de sang de diverses origines. Lorsqu'un phlébotome est dérangé au cours de son repas, il peut le compléter soit en piquant aussitôt le même hôte, expliquant ainsi certaines lésions multiples, soit en piquant un autre hôte. Les repas sanguins multiples sont plus souvent observés chez Phlebotomus papatasi (vecteur de LC à *L. major*), espèce très éclectique.

Le siège de prédilection des LC à *L. major*était les membres supérieurs et /ou inférieurs (61,7%) tandis que pour les LC à *L. tropica*, le visage était touché chez 70,3% des cas. Ce pourcentage est comparable à celui de la littérature [3]. Cette localisation peut être expliquée par le caractère nettement endophile du vecteur P.sergenti, ainsi que son attirance vers l'hôte humain qui semble dépendre de la production du dioxyde de carbone (CO2) mais également de l'odeur [14].

Le diamètre des lésions dans la L.C à *L.major* est en moyenne supérieur à celui des LC à *L. tropica*: 3,8 cm et 2,3 cm respectivement.

## Conclusion

Il ressort de l'analyse de nos résultats que les aspects cliniques des LC à *L. major* ou à *L. tropica*peuvent être confondus. Cependant, une localisation au visage serait plus évocatrice d'une LC à *L. tropica*, surtout si elle est unique, alors qu'une localisation aux membres serait plutôt évocatrice d'une LC à *L.major*. La PCR, en cours de mise au point dans le laboratoire de parasitologie mycologie de la faculté de médecine et de pharmacie de Casablanca, permettra certainement de vérifier ces données au Maroc et de mieux élucider cette nouvelle épidémiologie, étant donné que plusieurs espèces de LC peuvent être responsables de LC dans une même région. Le typage systématique des LC permettra de vérifier les résultats de cette analyse préliminaire et ainsi de proposer aux cliniciens une mise à jour des arguments cliniques en faveur d'une suspicion de LC.
